# Diffusion weighted cardiovascular magnetic resonance imaging for discriminating acute from non-acute deep venous Thrombus

**DOI:** 10.1186/s12968-019-0552-5

**Published:** 2019-07-08

**Authors:** Gang Wu, John Morelli, Yan Xiong, Xuanlin Liu, Xiaoming Li

**Affiliations:** 1 Department of Radiology, Tongji Hospital, Tongji Medical College, Huazhong University of Science and Technology, No.1095, Jiefang Avenue, Wuhan, 430030 Hubei China; 2 Department of Radiology, St. John’s Medical Center, Tulsa, OK USA

**Keywords:** Thrombus, MRI, Diffusion, Apparent diffusion coefficient

## Abstract

**Background:**

The importance of discriminating acute from non-acute thrombus is highlighted. The study aims to investigate the feasibility of readout-segmented diffusion weighted (DW) cardiovascular magnetic resonance (CMR) for discrimination of acute from non-acute deep venous thrombus (DVT).

**Methods:**

For this prospective study from December 2015 to December 2017, 85 participants (mean age = 53 years, age range = 34~74) with DVT of lower extremities underwent readout-segmented DW CMR. DVT of ≤14 days were defined as acute (*n* = 55) and > 14 days as non-acute (*n* = 30). DVT visualization on b = 0, b = 800, and apparent diffusion coefficient (ADC) images were assessed using a 4-point scale (0~3, poor~excellent). DW CMR parameters were measured using region of interest (ROI). Relative signal intensity (rSI) and ADC were compared between acute and non-acute DVT using a Mann Whitney test. Sensitivity and specificity for ADC and rSI were calculated.

**Results:**

ADC maps had higher visualization scores than b = 0 and b = 800 images (2.7 ± 0.5, 2.5 ± 0.6, and 2.4 ± 0.6 respectively, *P*<0.05). The mean ADC was higher in acute DVT than non-acute DVT (0.56 ± 0.17 × 10^− 3^ vs. 0.22 ± 0.12 × 10^− 3^ mm^2^/s, *P*<0.001). Using 0.32 × 10^− 3^ mm^2^/s as the cutoff, sensitivity and specificity for ADC to discriminate acute from non-acute DVT were 93 and 90% respectively. Sensitivity and specificity were 73 and 60% for rSI on b = 0, and 75 and 63% for rSI on b = 800.

**Conclusions:**

Readout segmented diffusion-weighted CMR derived ADC distinguishes acute from non-acute DVT.

**Trial registration:**

This study is retrospectively registered. Trial registration number: HUST-TJH-2015-146.

## Background

Deep venous thrombus (DVT) is a common disease [1, 2]. Venous ultrasonography is the first-line test and reference standard for detection of DVT. However, ultrasound is unable to accurately distinguish between acute and non-acute thrombus [3–6]. The treatment for acute thrombus requires anticoagulants with rapid onset. Thrombolytic therapy is recommended for acute thrombus, especially for newly-formed DVT. Few organized thrombi can be resolved by thrombolytic agents, so thrombolysis is not recommended for chronic DVT. Long term oral warfarin is required for non-acute DVT to prevent recurrent thrombosis. Thrombectomy is a good choice for newly-formed thrombus with large size, but not suitable for non-acute DVT due to adhesion of venous wall. Thus, the importance of discriminating acute from non-acute thrombus is highlighted.

Clinical determination of DVT age is often based on patient-reported symptoms and duration [7, 8]. Nearly one third of DVT are incorrectly staged using traditional diagnostic methods [7, 8]. Several promising imaging techniques have been developed to objectively assess thrombus age. For example, the age of thrombus can be inferred from its firmness. In this respect, ultrasound elastography imaging (UEI) has been reported to distinguish between acute and chronic thrombus by some authors, providing a useful adjunct to conventional ultrasound [9, 10]. However, UEI is highly operator dependent and is limited in its precision [11]. Some authors have evaluated cardiovascular magnetic resonance (CMR) to determine the age of DVT [12–14]. CMR direct thrombus imaging (CMRDTI) uses methemoglobin as an endogenous contrast agent [12, 15]. A study by Westerbeek et al. found that acute DVT demonstrated high T1 signal intensity on CMRDTI, which resolved within 3 months in 90% of patients [13]. Phinikaridou et al. used magnetic transfer (MT) and diffusion weighted (DW) imaging to visualize and detect protein composition of thrombus, thereby enabling determination of thrombus age in an animal model [14].

DW CMR has been widely used in clinical settings. Apparent diffusion coefficient (ADC) is the most widely used quantitative parameter [16]. We hypothesize that (1) diffusion in thrombus is restricted and (2) ADC decreases with thrombus age. The aim of the study is therefore to investigate the feasibility of segmented DW CMR for discriminating acute from non-acute DVT.

## Methods

This prospective study was approved by the university Institutional Review Board. Written informed consent was obtained from each participant before the study.

### Study participants

**Fig. 1:**
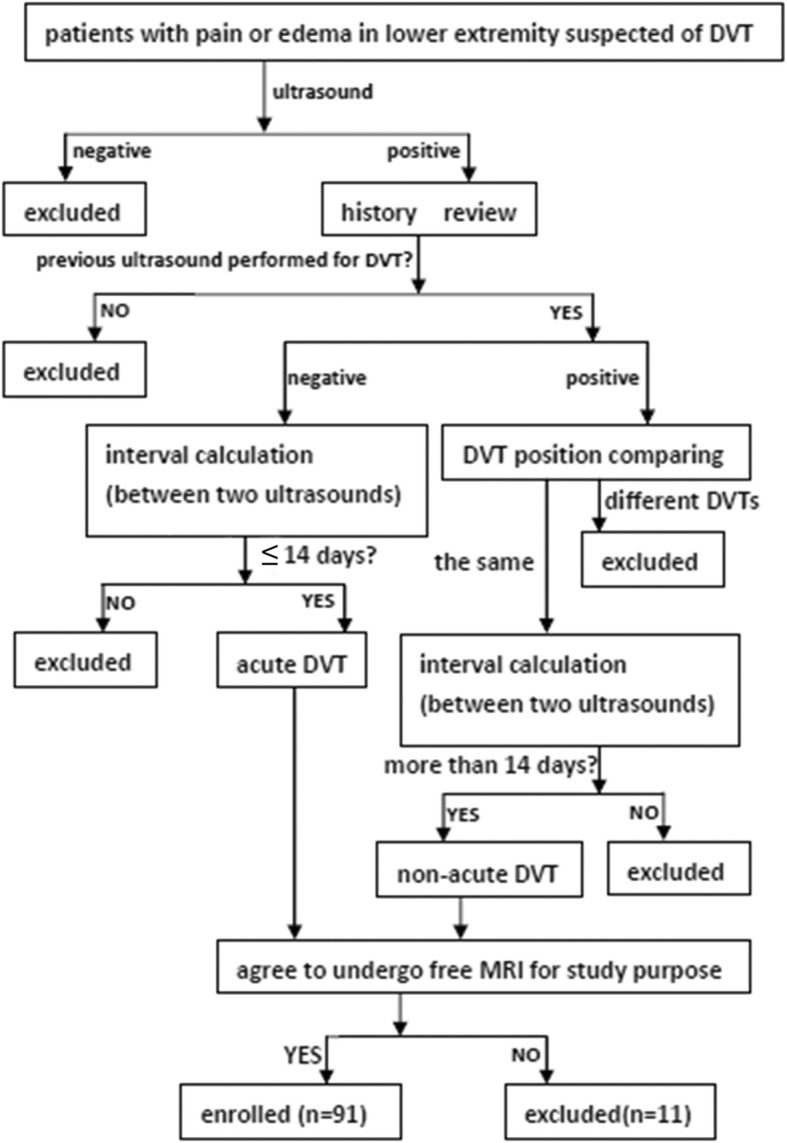
At least two ultrasounds were needed to confirm deep venous thrombosus (DVT) age. Acute DVT: less than or equal to 14 days; non-acute DVT: > 14 days

**Fig. 2:**
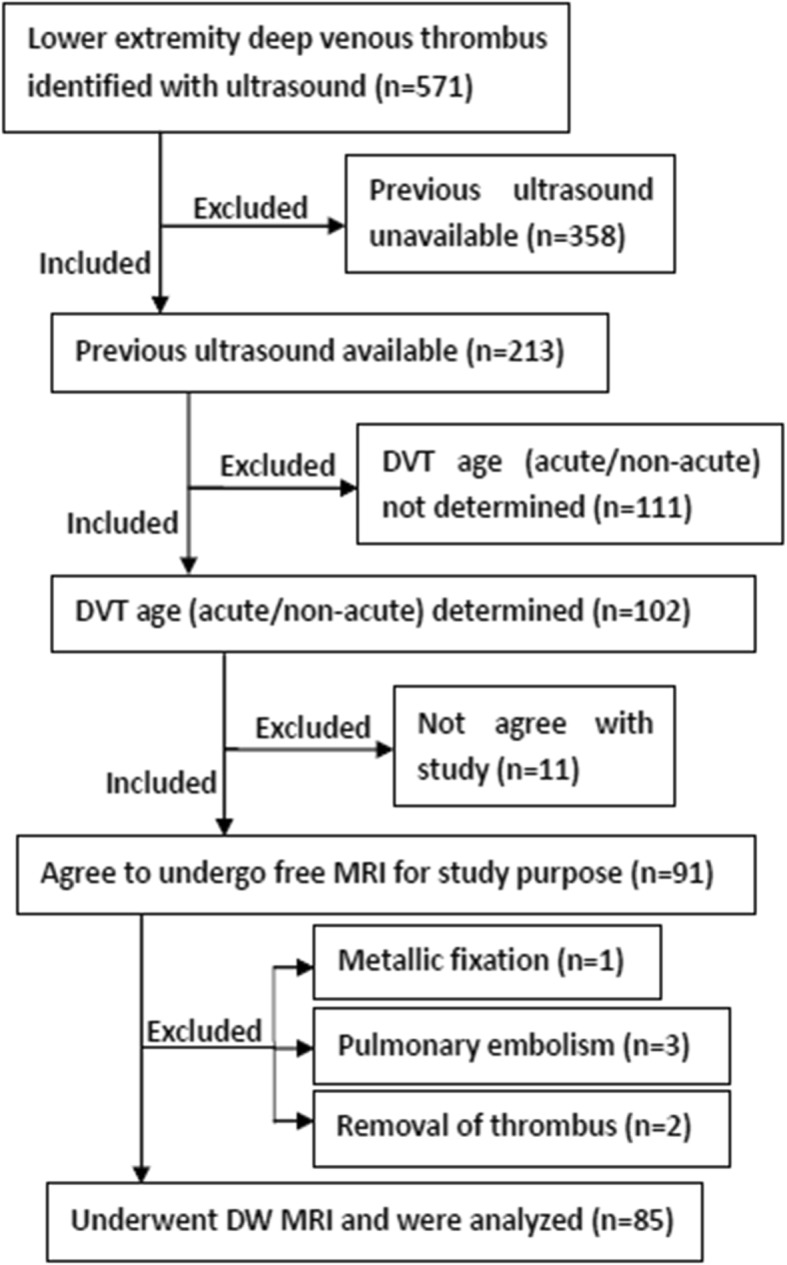
Flowchart indicating participants inclusion and exclusion. DVT age (acute/non-acute) could be determined if: 1) previous ultrasound negative, interval shorter than or equal to 14 days; or 2) previous ultrasound positive, interval > 14 days

Inclusion criteria were 1) participants with pain or edema in a lower extremity suspected of DVT (Fig. 1). Exclusion criteria were: (1) standard contraindications to CMR; (2) DVT removal with catheter-directed techniques; and (3) pulmonary embolism. From December 2015 to December 2017, an ultrasound radiologist with 15 years’ experience on ultrasound diagnosis of lower extremity vascular diseases classified study participants with acute (less than or equal to 14 days) versus non-acute DVT (greater than 14 days) according to Fig. 1. Thrombus age ≤ 14d were: 1) first ultrasound negative; 2) second ultrasound positive; 3) ultrasound interval less than or equal to 14 days. Thrombus age >14d were: 1) first ultrasound positive; 2) second ultrasound positive; 3) ultrasound interval more than 14 days. Inclusion and exclusion of participants are shown in Fig. 2.

### Ultrasound examinations

Standard venous ultrasound was performed by a radiologist specialized in peripheral vascular disease with 16 years of experience. We defined a thrombus present in the same location for > 14 days as a non-acute DVT and a thrombus present ≤ 14 days as an acute DVT.

### CMR examinations

The intervals between the ultrasound determination and the CMR examination were less than 24 h. All CMR examinations were performed on a 3 T whole-body scanner (Magnetom Skyra, Siemens Healthineers, Erlagen, Germany). Study participants were placed in supine position, feet-first. Two 18-element body coils were used to cover the regions of interest in the portions of the lower extremity.

Three-Dimensional sampling perfection with application optimized contrast using different flip angle evolutions (SPACE) was performed in the coronal plane for localization with the following parameters: TR/TE, 3200/100 ms; field of view, 44.8 cm × 28.7 cm; slice thickness, 1.8 mm; echo train length, 100; echo spacing, 4.04 ms; phase direction, head-foot; phase oversampling, 50%; slice number, 30 or more. After the full length of the thrombus was evaluated with SPACE, axial DW CMR was performed covering the center of the thrombus. Given the small size of the thrombus in axial dimensions, DW CMR with multiple readout segments was used. The main parameters of the readout-segmented DW CMR were as follows: TR/TE, 3000/70 ms; matrix size, 224 × 224; field of view, 22.4 cm × 22.4 cm or fit of leg size; slice thickness, 4 mm; slice number, 12–20; readout segments, 7 or more; b-values, 0 and 800 s/mm^2^; directions, 3; scanning time, 3~5 min. If more than one thrombus was identified by SPACE, the thrombus with the greatest length was studied for the sake of simplicity.

### Data analysis

Two radiologists with 8 and 11 years’ experience on CMR diagnosis of vascular diseases blinded to all information evaluated DVT visualization on b = 0, b = 800 and ADC images in consensus. A subjective score was given according to a 4-point scale: 0 = poor depiction of thrombus caused by artifacts or other image degradation with ROI assessment not possible; 1 = moderate depiction of thrombus with unclear boundaries but ROI assessment possible; 2 = good depiction of thrombus, adequate thrombus-to-background contrast, clear boundary; 3 = excellent depiction of thrombus, excellent thrombus-to-background contrast, clear boundary. The signal type of DVT on DW CMR source images was also determined by the two readers in consensus: hyperintensity was defined as brighter than background, isointensity as close to background, and hypointensity as darker than background.

Two radiologists with 11 and 9 years’ experience on imaging diagnosis of vascular diseases blinded to all information measured the signal intensity (SI) of thrombus, normal muscle and background air on DW source images, and ADC values on ADC maps by drawing ROI. ROI on muscles avoided edema areas. The standard deviation of air SI was defined as noise. The relative signal intensity (rSI) was obtained by calculating the ratio of thrombus SI to muscle SI. rSI_0_ and rSI_800_ represented rSI on b = 0 and b = 800 images, respectively. The signal-to-noise ratio (SNR) was obtained by calculating the ratio of thrombus SI to noise.

### Statistical analysis

All data statistical analysis was performed with SPSS (version 22.0, Statistical Package for the Social Sciences (SSPS) International Business Machines, Inc., Armonk, New York, USA). The Wilcoxon signed-rank test was used to compare the visualization scores between different maps. The Mann Whitney test was used to compare the visualization scores between acute and non-acute DVT. Intra-class correlation coefficient (ICC) was calculated to determine the inter-reader viability in measurement of parameters. ICC above 0.8 was considered excellent inter-reader reproducibility, thus data from two readers were averaged. A Mann Whitney test was used to determine the difference in DW CMR parameters between acute and non-acute DVT. A non-paired student’s t test was used to compare study participant age. A Chi square test was used to identify gender differences. Receiver operating characteristic (ROC) curves were constructed to evaluate the ability in discriminating acute from non-acute DVT. Cutoff corresponding with the highest Youden Index was chosen. *P* values less than 0.05 was considered significant difference.

## Results

From December 2015 to December 2017, 85 study participants (53 years, range = 34~74) underwent CMR, including 55 participants with acute DVT (male: female = 23: 32, average age = 53 years) and 30 participants with non-acute DVT (male: female = 11: 19, average age = 52 years). There was no difference in age or gender between the two groups (*P*>0.05). Possible causes of DVT and correlated diseases are listed in Table 1. Exclusion of participants for any reason is shown in Fig. 2.

**Table 1:** Study participant demographics with causes of and predisposing disorders for DVT

	Acute DVT (*n* = 55)	Non-acute DVT (*n* = 30)	Total (*n* = 85)
Male: female	23:32	11:19	34:51
Mean age, age range	53, 37~74	52, 34~71	53, 34~74
Causes			
Ttumor	13	12	25
Operation	19	5	24
Limb immobility activity	9	5	14
Others	14	8	22
Correlated diseases			
Cancer	22	16	38
Cerebral infarction	5	3	8
Myelitis	4	2	6
Rheumatoid diseases	3	2	5
Trauma	7	1	8
Pregnancy	2	0	2
Infection	2	1	3
Others	10	5	15

*DVT* deep venous thrombus

The DVT visualization scores are shown in Table 2. 53% of the b = 0 images (45/85) provided excellent depiction of DVT and 45% (38/85) a good depiction. 41% of the b = 800 images (35/85) provided excellent depiction and 53% (45/85) good. 68% of the ADC maps (58/85) provided excellent depiction and 32% (27/85) good depiction. The average scores for b = 0, b = 800 images and the ADC maps are shown in Table 2. Visualization scores were higher with the ADC maps than with the b = 0 and b = 800 images (*P* < 0.05). Scores did not differ between acute and non-acute DVT (*P* > 0.05).

**Table 2:** Visualization of DVT was scored according to the following scale: 0 = poor depiction of thrombus; 1 = moderate depiction of thrombus; 2 = good depiction of thrombus; 3 = excellent depiction of thrombus. The Wilcoxon signed-rank test was used to compare the visualization scores of DVT between different maps. The Mann Whitney test was used to compare the visualization scores between acute and non-acute DVT

	Poor(0)	Moderate(1)	Good(2)	Excellent(3)	Mean ± SD		P
b = 0 map	0	2	38	45	2.5 ± 0.6	ADC vs. b = 0	<0.001
b = 800 map	0	5	45	35	2.4 ± 0.6	ADC vs. b = 800	<0.001
ADC map	0	0	27	58	2.7 ± 0.5	b = 0 vs. b = 800	0.02
	b = 0 map			b = 800 map		ADC map	
Acute	2.6 ± 0.6			2.4 ± 0.6		2.7 ± 0.5	
Non-acute	2.4 ± 0.5			2.2 ± 0.6		2.7 ± 0.5	
P	0.27			0.09		0.82	

The mean SNR was 96.4 ± 65.5 for thrombus at b = 0 map, and the SNR range was 18.8~254.5. The mean SNR was 134.3 ± 101.4 for thrombus at b = 800 map, and the SNR range was 21.2~297.1.

**Fig. 3:**
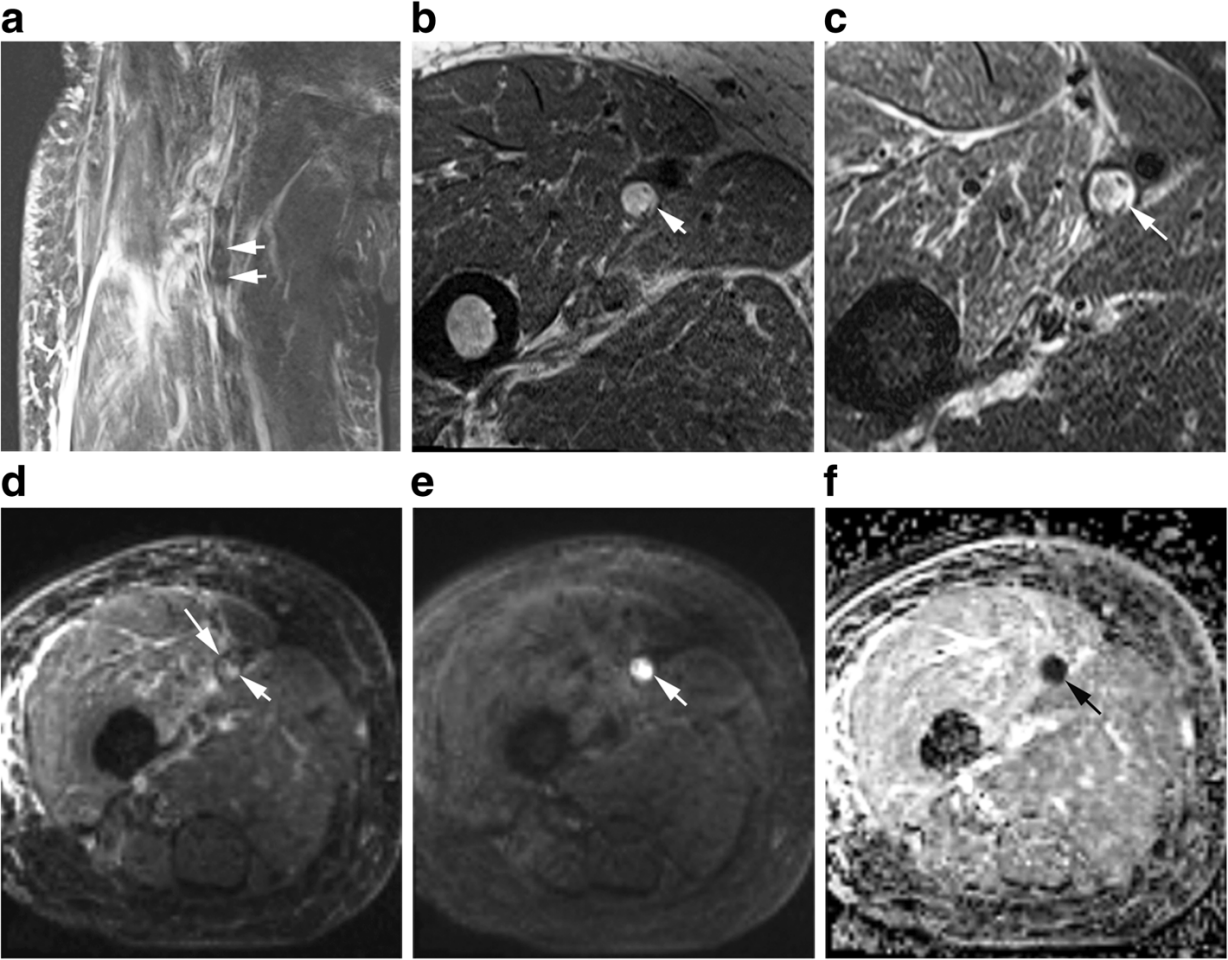
Fifty-seven years old female participant with lower extremity edema. This acute DVT was heterogeneously hypointense on SPACE (**a**, arrows), mixed hyper- and hypointense on T1 (**b**, arrow) and T2 (**c**, arrow) weighted images. The DVT signal on b = 0 image (**d**, arrow) is similar to that on T2 images. On the b = 800 image (**e**, arrow), the thrombus was extremely high signal. On the apparent diffusion coefficient (ADC) map (**f**, arrow), the thrombus was darker than background. E and F provided adequate thrombus-to-background contrast, but D did not

**Fig. 4:**
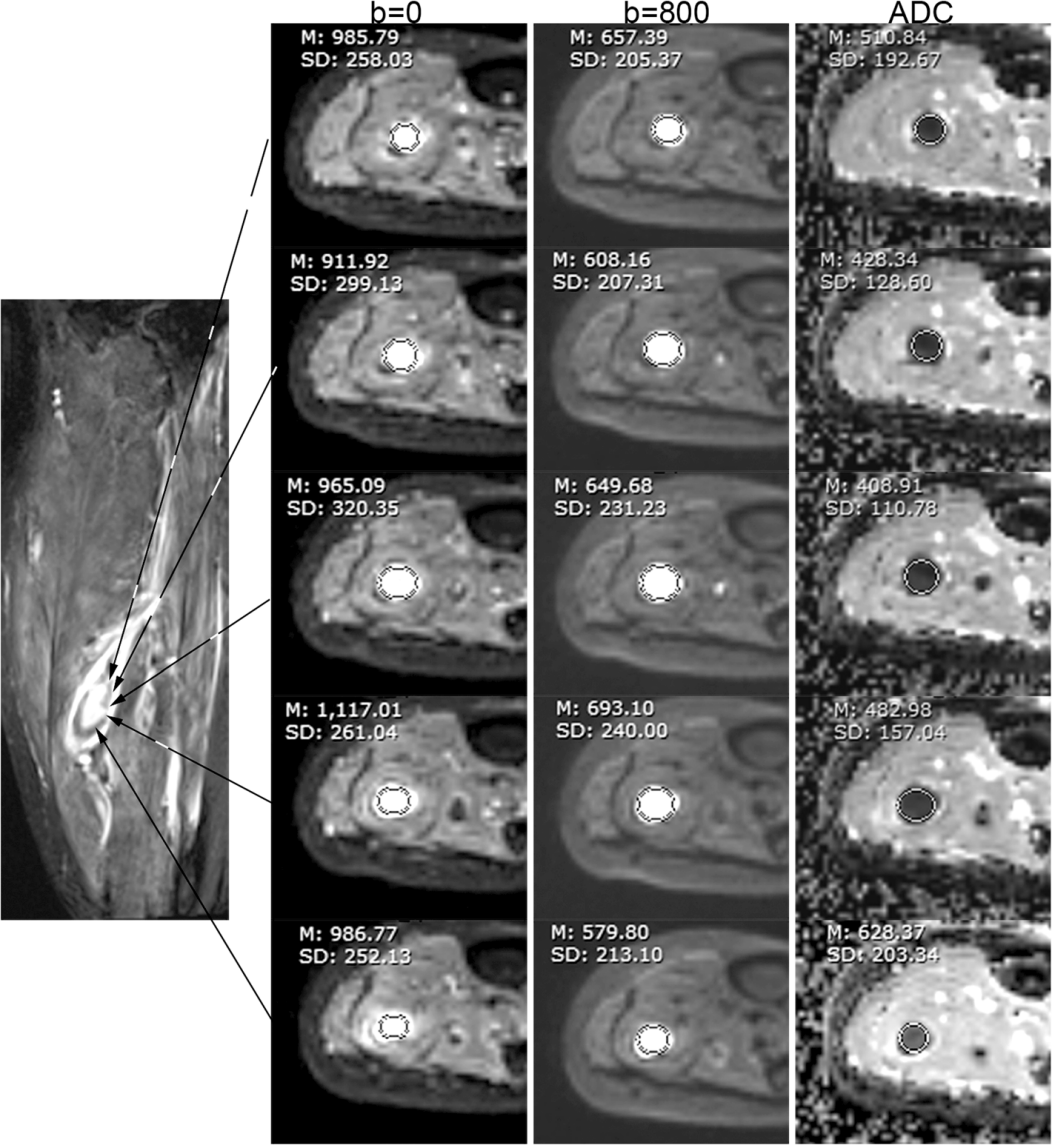
Sixty-five years old male participant with edema and pain of calf. From left to right, the columns are an SPACE, b = 0, b = 800, and ADC map. On SPACE, this acute DVT was centrally hyperintense and peripherally hypointense. On the b = 0 and 800 images, the DVT was hyperintense. The DVT was hypointensity on ADC map. All values acquired in the regional of interest (ROI) analysis indicated acute DVT

All DVT demonstrated low ADC values compared to adjacent tissue (see Table 3, Figs. 3, 4). Most DVT had adequate thrombus-to-background contrast (see Figs. 3, 4). ICC for DW CMR parameters are shown in Table 4. As all ICC above 0.8, the data from two readers were averaged.

**Table 3:** Appearance of acute and non-acute DVT on DW CMR

	b = 0 s/mm^2^			b = 800 s/mm^2^			ADC map		
	Hyperintensity	Isointensity	Hypointensity	Hyperintensity	Isointensity	Hypointensity	Hyper	Iso	Hypo
Acute	33	3	19	34	4	17	0	0	55
Non-acute	5	2	23	6	3	21	0	0	30
Total	38	5	42	40	7	38	0	0	85

Hyper, ADC value higher than background; iso, ADC value close to background; hypo, ADC value lower than background

*DVT* deep venous thrombus

**Table 4:** DW CMR parameters were compared between acute and non-acute DVT. Relative signal intensity was the ratio of thrombus SI to normal muscle SI. The units are 10^−3^ mm^2^/s for ADC. Sensitivity and specificity in identifying acute DVT from non-acute DVT were obtained using the cutoff given in table

	ICC	Acute vs. non-acute	P	Cutoff	Sensitivity, 95%CI	Specificity, 95%CI
rSI_0_	0.91	2.0 ± 1.3 vs. 1.0 ± 0.8	0.01	0.95	73% (40/55), 59%~ 83%	60% (18/30), 41%~ 77%
rSI_800_	0.89	3.1 ± 1.9 vs. 1.6 ± 1.3	0.02	1.55	75% (41/55), 61%~ 85%	63% (19/30), 44%~ 79%
ADC	0.89	0.56 ± 0.17 vs. 0.22 ± 0.12	<0.001	0.32	93% (51/55), 82%~ 98%	90% (27/30), 72%~ 97%

*DVT* deep venous thrombus, *SI* signal intensity, *ICC* intra-class correlation coefficient, *CI* confidence interval, *rSI*_*0*_ relative signal intensity on b = 0, *rSI*_*800*_ relative signal intensity on b = 800

### Comparison between acute and non-acute DVT

**Fig. 5:**
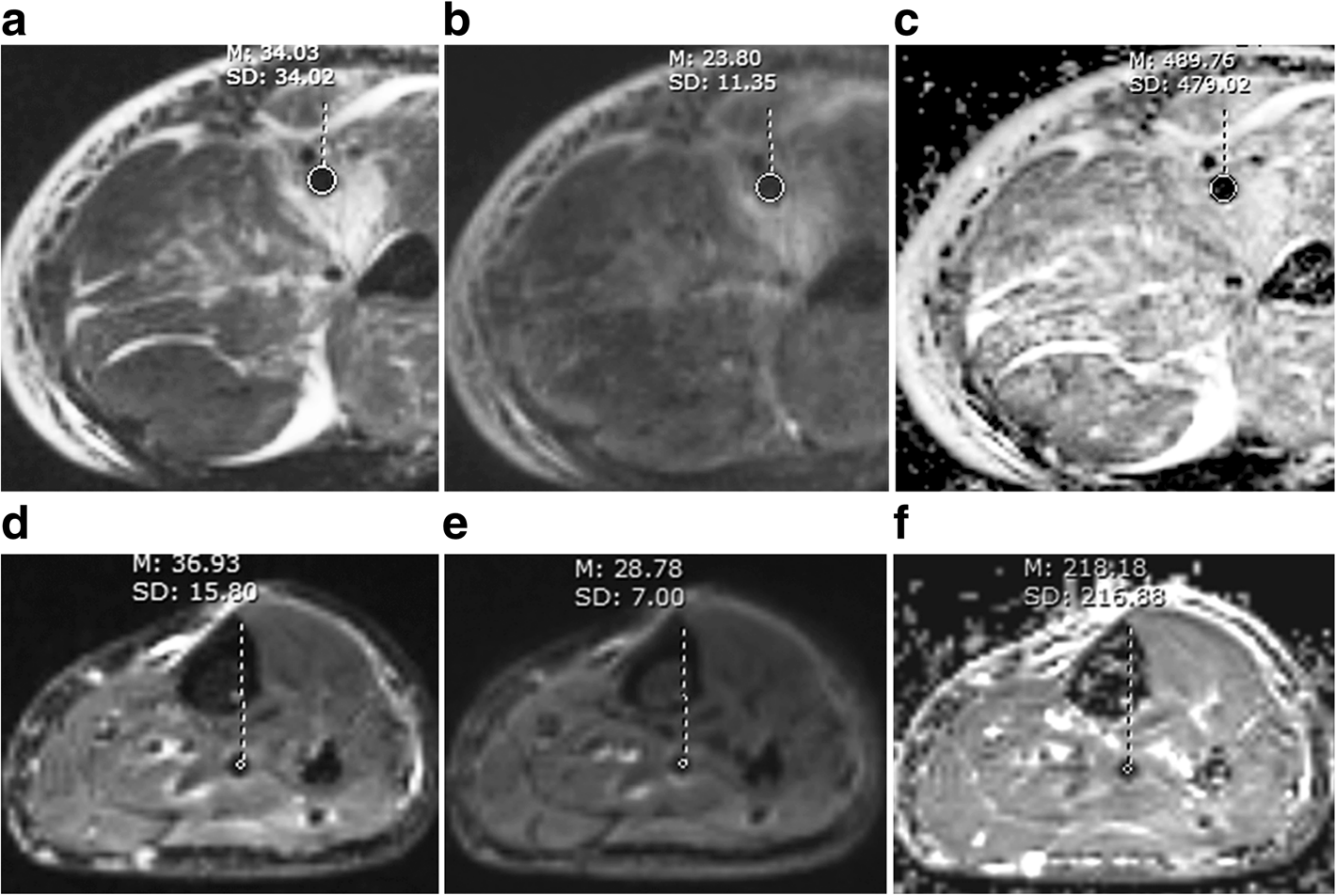
A comparison between acute and non-acute DVT. Acute thrombus (first line) and non-acute thrombus (second line) were hypointense on b = 0 (**a**, **d**), b = 800 s/mm^2^ (**b**, **e**) image. The signal intensity was about the same for acute and non-acute thrombus, while ADC values differed greatly (**c**, **f**). Mean ADC of this acute thrombus was above the cutoff of 0.32 × 10^− 3^ mm^2^/s, while that of non-acute thrombus was below the cutoff

Signal types of acute and non-acute DVT are compared in Table 3. The rSI_0_ and rSI_800_ were higher in acute DVT versus non-acute DVT (P<0.05, Table 4). The acute DVT mean ADC was higher than that of non-acute DVT (0.56 ± 0.17 × 10^− 3^ mm^2^/s vs. 0.22 ± 0.12 × 10^− 3^ mm^2^/s, P<0.001). Figure 5 is a comparison of acute and non-acute DVT. Both are hypointensity on DW source images. They have similar signal intensity, but ADC values differ greatly.

**Fig. 6:**
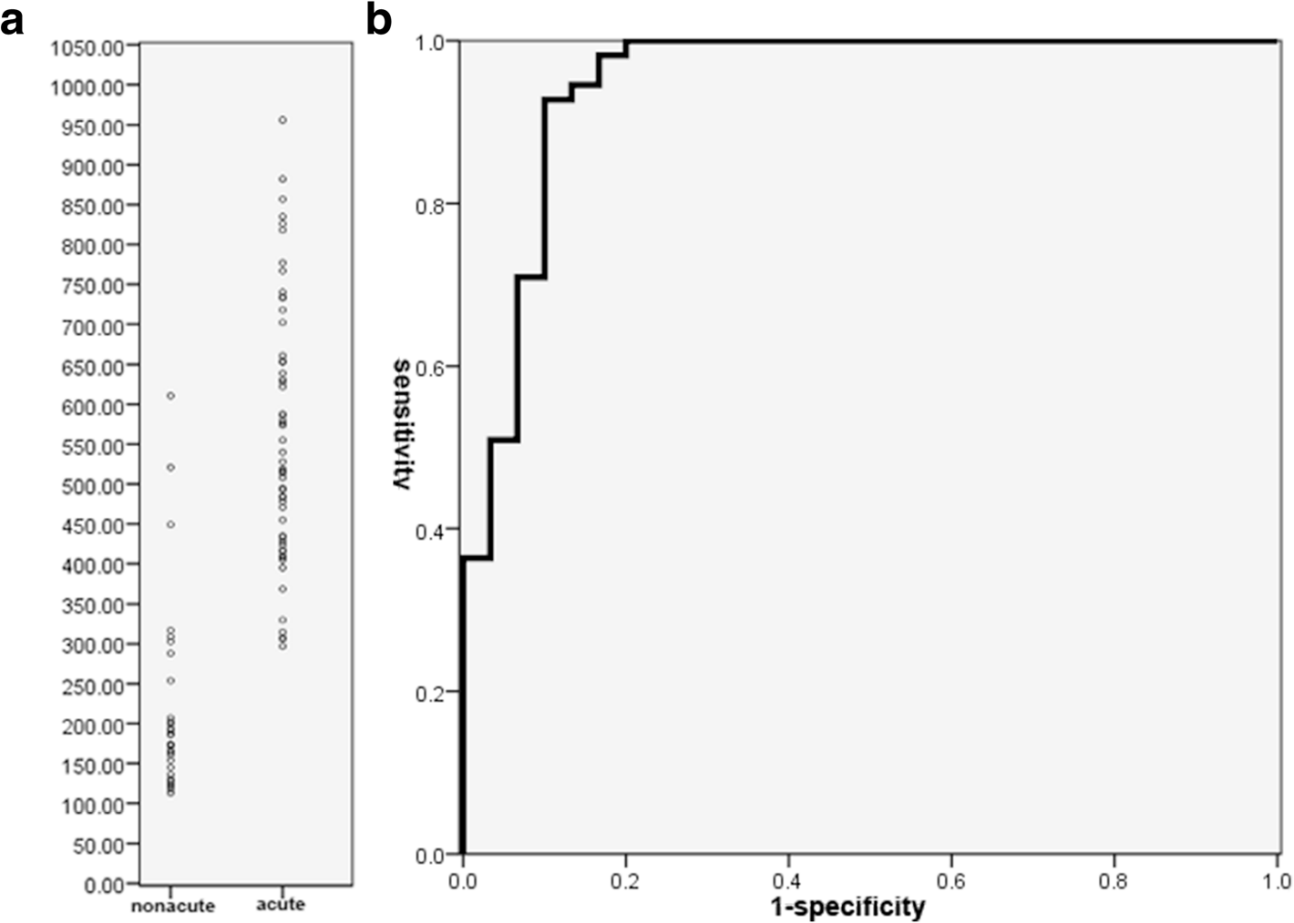
ADC distribution and ROC curve. Overlap of ADC could be seen between acute and non-acute DVT (**a**). Area under curve was 0.948 for ADC in discriminating acute from non-acute DVT (**b**)

Figure 6 shows the distribution of ADC values of acute and non-acute DVT. When using 0.32 × 10^− 3^ mm^2^/s as the cutoff, the sensitivity and specificity for ADC in discriminating acute from non-acute DVT were 93% (51/55, 95% confidence interval, 82%~ 98%) and 90% (27/30, 95% confidence interval, 72%~ 97%) respectively. The cutoffs, sensitivities and specificities for rSI_0_ and rSI_800_ are shown in Table 4.

## Discussion

Our underlying hypothesis was that ADC decreases with thrombus age. We therefore investigated the use of readout-segmented DW CMR to discriminate acute (≤ 14 days) from non-acute (> 14 days) DVT. Our primary findings were: 1) ADC was different between acute and non-acute DVT (0.56 ± 0.17 × 10^− 3^ mm^2^/s vs. 0.22 ± 0.12 × 10^− 3^ mm^2^/s, respectively, *P* < 0.001); 2) ADC values had 93% sensitivity and 90% specificity for discriminating acute from non-acute DVT.

DVT visualization scores were highest with ADC maps, because the thrombus-to-background contrast on ADC map was always adequate. DVT ADC was lower than that of muscle background in every case. In some cases, thrombus signal was close to background on DW CMR source images, thus reducing conspicuity.

Free water content is less in non-acute DVT compared with acute DVT, because water content decreases with thrombus age [17, 18]. The space where water molecular can diffuse is narrower in non-acute thrombus versus acute thrombus, as DVT becomes more compact with age [19]. Thus ADC of non-acute DVT should be lower than that of acute DVT. Our study supports this hypothesis.

ADC had acceptable sensitivity and specificity in the DVT discrimination. The signal of thrombus on DW CMR varied and could be low, intermediate, or high signal. DVT signal intensity on b = 0 map depends on both water content and paramagnetic composition. Paramagnetic methemoglobin content gradually increases with thrombus age due to oxidation of hemoglobin, peaking at about 7~10 days and then gradually decreasing due to phagocytosis [20]. Thus, acute DVT with age of about 7~10 days might be expected to demonstrate low T2 signal due to the resulting T2 shortening. In fact, some acute DVT in our study were low signal intensity on b = 0 images. Most non-acute DVT had low signal intensity on b = 0 images, likely secondary to low water content.

We used readout-segmented DW CMR instead of traditional DW CMR due to the small axial dimensions of thrombus, because traditional DW CMR did not provide adequate spatial resolution, and was frequently degraded by susceptibility artifacts in lower extremity. By using segmented readouts, readout-segmented DW CMR is able to overcome some of the aforementioned limitations. For example, increased matrix size and/or smaller field of view enable higher in-plane resolution. Unfortunately, readout-segmented DW CMR does require longer acquisition times versus single shot DW CMR. Long scan times typically result in more patient induced motion. Fortunately, our DW CMR was 2D-acquisition performed in the transverse plane, perpendicular to the long axis of leg, so was not susceptible to leg motion. The acquisition time increases with number of readout segments, so the selection of the number of readout segments must balance spatial resolution and acquisition time.

Main clinical constraints that prevented readout-segmented DW CMR may be as follows: 1) the availability of CMR is inadequate in some centers; 2) some patients have contraindications to CMR examination; 3) readout-segmented DW CMR is not as conventional as fast spin echo T1 or T2, so radiologists need training of scan and measurement. However, all the constraints could be overcome with the development of hardware and software.

Our study has several limitations. First, thrombectomy was not performed for the participants studied, so DVT could not be pathologically assessed to establish the actual water and methemoglobin content. Second, the DVT age classification in our study is binary (≤14 days and >14 days). ADC values may in reality differ among hyper-acute, acute DVT, sub-acute, and chronic DVT. Finally, the sample size is relatively small, especially for non-acute DVT. Larger, multi-center studies may help further clarify these results.

## Conclusions

In conclusion, using readout-segmented DW CMR, ADC values were greater for acute DVT (≤14 days) compared to non-acute DVT (>14 days). ADC values may be helpful for determining the age of DVT, and could potentially be used to help triage therapy in selected patients.

## Data Availability

The datasets used and/or analysed during the current study are available from the corresponding author on reasonable request.

## Abbreviations

ADC: Apparent diffusion coefficient
CMR: Cardiovascular magnetic resonance
CMRDTI: CMR direct thrombus imaging
DVT: Deep venous thrombus
DW: Diffusion weighted
ICC: Intra-class correlation coefficient
MT: Magnetization transfer
ROC: Receiver operator characteristics
ROI: Region of interest
rSI: Relative signal intensity
SI: Signal intensity
SNR: Signal-to-noise ratio
SPACE: Sampling perfection with application optimized contrast using different flip angle evolutions
UEI: Ultrasound elastography imaging
